# Effect of Case Management Interventions for Patients with Substance Use Disorders: A Systematic Review

**DOI:** 10.3389/fpsyt.2017.00051

**Published:** 2017-04-06

**Authors:** Louise Penzenstadler, Ariella Machado, Gabriel Thorens, Daniele Zullino, Yasser Khazaal

**Affiliations:** ^1^Geneva University Hospitals, Geneva, Switzerland; ^2^Faculty of Medicine, Geneva University, Geneva, Switzerland; ^3^Research Center, Montreal University Institute of Mental Health, Montreal, QC, Canada

**Keywords:** case management, assertive community treatment, substance use disorder, substance abuse, alcohol use disorder

## Abstract

**Background:**

Substance use disorder (SUD) is an important health problem that requires a complex range of care because of the chronic nature of the disorder and the multiple psychosocial problems involved. Current outpatient programs often have difficulties in delivering and coordinating ongoing care and access to different health-care providers. Various case management (CM) models have been developed, first for patients in other psychiatric domains and then for patients with SUD, in order to improve treatment outcomes.

**Aim:**

This paper aims to assess the effectiveness of CM for patients with SUD.

**Methods:**

We performed a systematic review of CM interventions for patients with SUD by analyzing randomized controlled studies published on the subject between 1996 and 2016 found on the electronic database PubMed.

**Results and conclusion:**

Fourteen studies were included in the analysis. Differences between studies in outcome measures, populations included, and intervention characteristics made it difficult to compare results. Most of these studies reported improvement in some of the chosen outcomes. Treatment adherence mostly improved, but substance use was reported to decrease in only a third of the studies. Overall functioning improved in about half of the studies. The heterogeneity of the results might be linked to these differences between studies. Further research is needed in the field.

## Introduction

Substance use disorders (SUDs), which include drug abuse, problematic drug use, drug misuse, and substance misuse, are an important health problem (1). Persons with SUDs are characterized by multiple social and medical needs and are often known for their difficulty in engaging in treatment, partly because access to treatment facilities is limited (2). The chronicity and relapsing nature of SUD, as in other psychiatric disorders, entails frequent hospitalizations (3) and readmissions.

Patients presenting both severe mental illnesses and SUD are typically hospitalized more often than are non-substance users (4, 5). Patients presenting this double diagnosis also have more difficulties entering alcohol and drug outpatient clinics than patients with only SUD (6). This group of patients seems to have less access to aftercare services (7) and higher use of acute services, such as emergency room treatment and hospital services (8).

The period after discharge is characterized by a high risk of relapse, with most cases occurring within the first week of inpatient treatment (9). There is also an important risk of drug-related death (either accidental or intended) following a longer period of abstinence because of lower drug tolerance (10, 11). These patients have multiple psychosocial problems for which they need support. Patient needs often remain unmet in current outpatient treatment programs (2), although the provision of help with legal advice, basic needs, and family services may improve patients’ psychosocial functioning. Treatment continuity has been related to higher overall abstinence rates (12, 13) and less frequent readmissions to hospital units (14). Between hospital and community care, treatment continuity is supposed to improve comprehensive support for patients.

Different strategies have been developed to improve treatment adherence and drug-related outcomes (15); among them, case management (CM) has been identified as potentially beneficial as suggested in early clinical studies (16). The definition of CM and its practice varies from place to place. In general, CM can be defined as a “coordinated integrated approach to service delivery, ongoing supportive care and help to access resources for living and functioning in the community” (17). This approach has been widely implemented in many different areas, such as insurance programs, education, and health care.

Given the complex, chronic, and relapsing nature of mental health disorders and SUDs, they require a broad and continuous approach such as can be offered by CM (17). Since the 1980s, this practice has been adapted for persons with SUD (18), but to date, only a few studies have described CM models for persons with SUD in Europe.

The aim of this study was thus to assess the effectiveness of CM for patients with SUD. We searched for published articles in which clinical CM was described for patients with SUD to help maintain treatment continuity and coordinate care after a patient was discharged from hospital or prison (transitional CM) or when a patient entered a treatment program.

## Method

The electronic database PubMed was searched for empirical studies published between January 1996 and May 2016. The following keywords were used: “case management” AND “addiction”; “case management” AND “substance use disorder”; “case management” AND “substance abuse.” The inclusion criteria were as follows: randomized controlled trial, adult participants over the age of 18 years with SUD, and a CM intervention compared to treatment as usual (TAU).

## Results

**Figure 1:**
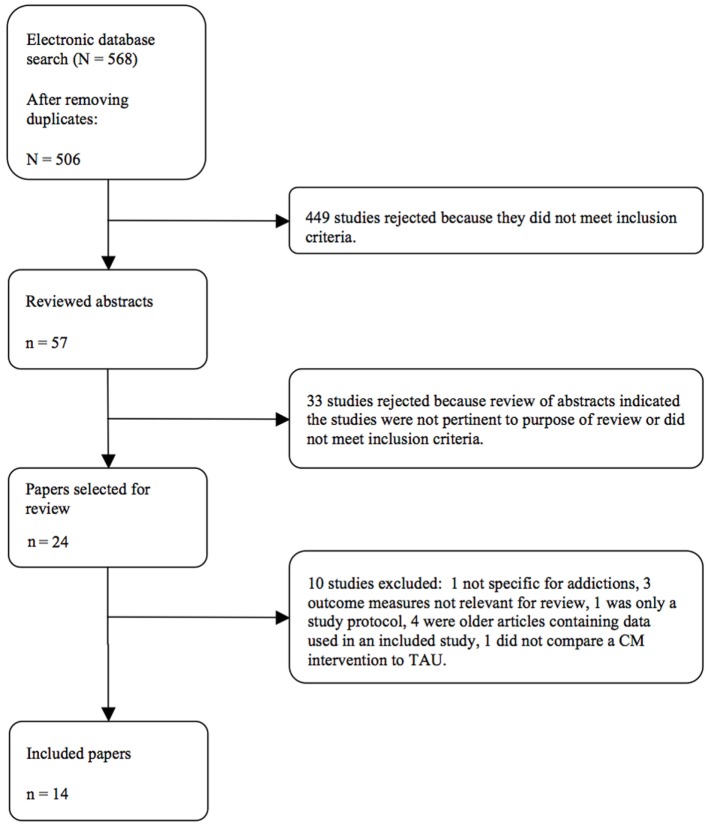
**Study flow diagram**.

After checking for the inclusion criteria and for duplicates, we analyzed 14 studies (Figure 1). Details about the included studies are described in Table 1. One paper (19) was reviewed but excluded. It compared assertive community treatment to another form of CM intervention. In absence of a TAU comparison group, the study was not included.

**Table 1:** **Characteristics of the included studies**.

Reference, country	Target population	Number of subjects	Control intervention	CM interventions/dose of CM	Outcome measures	Follow-up	Results
Guydish et al. (30), USA	Drug-involved women offenders on probation or awaiting probation who were willing to enter a substance abuse treatment program	*N* = 183 IG: *n* = 92; CG: *n* = 91	TAU = standard probation	12 months of PCM involving uniform assessment procedures, a therapeutic and advocacy orientation, treatment planning, counseling, and home visits. Dosage: at least two contacts per month (visit or phone)	ASI, BDI, BSI, Social Support Evaluation List, service utilization, arrest during 12 months of face time with CM	6 and 12 months	Proportion of women enrolled in SUD treatment or incarcerated was not statistically different for both groups. All other measures were not statistically different between groups. At 6 months, 53.6% of PCM participants met face-to-face with case manager once or more and at 12 months 43.5% did. In CG, this was 11.6 and 8.5%, respectively. This shows that the dosage was often a lot less than twice a month, as described in the intervention. The participants who had two or more contacts with case manager were more likely to have lower ASI rates and lower social severity rates
Essock et al. (20), USA	Alcohol and illicit drug users with a co-occurring major psychotic disorder, who had high service use in the past 2 years, were homeless or unstably housed, and had poor living skills	*N* = 198 IG: *n* = 99; CG: *n* = 99	Standard clinical CM: comprehensive assessment, individual MI, group treatments, and stage-wise interventions	Three years of community-based assertive CM treatment: direct substance abuse treatment by case managers and comprehensive assessment, individual MI, group treatments, and stage-wise interventions. Case managers had half the patient load that they had for CG	Substance use (days of use, ASI, toxicology screens) structured interview and rating scales assessed by case manager; hospitalization rates; Quality of Life Interview; CM dosage: contacts per month with case manager	Every 6 months	Participants in both treatment conditions improved over time in multiple outcome domains, and few differences were found between the two models. Decreases in substance use were greater than would be expected given time alone. At the site that had higher rates of institutionalization, clients who received standard CM were more likely to be institutionalized. However, in the site that had lower rates of institutionalization, no differences in the rate of institutionalization were found between the two treatment conditions. At one site, the IG received a significantly higher dose (time and activities) of services than did the CG. At the other site, the difference was not significant. Integrated treatment can be successfully delivered either by assertive community treatment or by standard clinical CM
Huber et al. (21), USA	Drug or alcohol users who were diagnosed with substance abuse disorder and enrolled at a substance abuse treatment facility	*N* = 598 IG: *n* = 437; CG: *n* = 149	Standard drug abuse treatment	Community-based comprehensive CM intervention: 12 months of CM interventions consisted of four CM conditions with a case manager working as a member of drug-treatment staff (inside), a case manager from an outside social service agency (outside), or a case manager using computerized telecommunication (telecom). CG received standard drug abuse treatment. Five types of CM interventions were assessing, individual solution planning, referral, advocating, and conferencing	CM dosage, ASI	3, 6, and 12 months	Clients who engaged (actively participated) in CM were less likely to have legal and family issues, but more likely to have a chronic medical condition at baseline. Dosage factors differed significantly across treatment conditions. In general, dose was significantly related to outcomes in the legal and family domains
Lindahl et al. (23), Sweden (EU)	Court-ordered substance abuse patients	*N* = 34 IG: *n* = 13; CG: *n* = 21	TAU	Six months of CM intervention: case managers offered assessment, transitional care, support of referral services, and intervention to avoid crisis	Substance use (ASI, AUDIT, AUDRUG, SIP, days of alcohol used); psychological functioning; involuntary care (coercive measures); number of days in institutional or hospital care was measured	6 and 12 months after discharge	More patients from the CM group were abstinent compared with those in the CG at the first follow-up at 6 months (46 vs. 14%, *p* < 0.051).Patients in the CM group did not have more contact with health and social services (92%) compared with those in the CG (76%) (*p* = 0.23), nor did they have more medical-assisted treatment (*p* = 0.46) or institutional/inpatient care (*p* = 0.27) to a higher degree than patients in the CG.CM interventions were well received by the patients with no dropout during intervention. Patients with the support of a case manager seemed to sustain abstinence to a higher degree compared with TAU, but no differences were detected regarding use of care. A subgroup analysis showed that patients with continuous drug abuse had access to care from both social welfare and hospital care systems
Morgenstern et al. (22), USA	Women with SUD receiving temporary assistance for needy families; not psychotic, under methadone treatment or seeking methadone treatment, or already in treatment program	*N* = 302 IG: *n* = 161; CG: *n* = 141	TAU, which was standard substance abuse screening and referral system within welfare department	ICM intervention: CM services were provided throughout the 15-month follow-up period; assessment, planning, motivational enhancement, treatment coordination, peer support, and crisis management. If needed, case managers provided home visiting services. Contact was adapted to needs from daily to two visits per month	Substance use (ASI, toxicology screen). Treatment attendance. Treatment engagement. Treatment retention rate	3, 9, and 15 months; 24 months (article 28)	ICM clients had significantly higher levels of substance abuse treatment initiation, engagement, and retention compared with CG clients. In some cases, ICM treatment attendance rates were double those of CG rates. Additionally, almost twice as many ICM clients were abstinent at the 15-month follow-up compared with CG clients (*p* < 0.0025). After 24 months, abstinence rates were higher in the ICM group than they were for usual care. Additionally, there were greater odds of being employed full time
Morgenstern et al. (31): 24-month outcome							
Morgenstern et al. (29), USA	SUD welfare applicants without acute psychotic symptoms and not more than one hospitalization for mental health problems in the last year	*N* = 394 (66% men) IG: *n* = 221; CG: *n* = 173	Usual care	CCM: continuity of care intervention focused on engaging clients in drug treatment, linking to needed ancillary services, and fostering transition to employment. Biweekly visit at treatment center and regular contact in office or by phone	Employment outcomes (days of employment and percentage of full-time employment), abstinence rates, treatment attendance	1-year follow-up	Overall, men were more likely to work than women. There was no difference between groups. CCM increased women’s employment over time. Among women only, greater SUD treatment attendance and abstinence in the first 6 months of CCM predicted higher rates of later employment
Plater-Zyberk et al. (27), ON, Canada	Patients enrolled in a methadone maintenance treatment program	*N* = 1,704 IG: *n* = 396; CG: *n* = 1,308	TAU: standard outpatient treatment	Clinical CM: duration and frequency varied according to clients’ needs	Drug-positive urine samples, missed daily methadone doses, missed methadone physician appointments	3 months	The IG demonstrated statistically significant improvement in all three measures of the methadone maintenance treatment program. Less drug-positive urine: 15.4% relative reduction. Fewer missed daily methadone doses: 2% relative reduction. Fewer missed appointments with the methadone physician: 40% relative reduction
Prendergast et al. (28), USA	Correction population who were enrolled in a drug-treatment program within a correctional institution (prison, work release, community correctional facility) in four states	*N* = 812 (men and women) IG: *n* = 412; CG: *n* = 400	Standard referral/services (SR group)	TCM using the SBCM model: strengths assessment, conference call 1 month prior to release, community sessions. After release, weekly sessions for 3 months, followed by 3 monthly follow-up contacts for any client needing additional help	SUD treatment services, other social services, drug use, alcohol use, arrest, HIV risk behavior	3 and 9 months following release from prison	There were no significant differences between parolees in the TCM group and the SR group on outcomes related to participation in drug abuse treatment, receipt of social services, or drug use, crime, and HIV risk behaviors. For specific services (e.g., residential treatment, mental health), although significant differences were found for length of participation or for number of visits, the number of participants in these services was small and the direction of effect was not consistent
Rapp et al. (24), USA	Substance abusers seeking treatment; not psychotic and not only alcohol use disorder	*N* = 678. SBCM: *n* = 222 One session of MI: *n* = 226; CG: *n* = 230	Standard care at a centralized intake unit	SBCM: assessing, individual solution planning, referral, advocating, and conferencing. Up to five sessions of SBCM. MI: clarify motivation, reinforce treatment-seeking behaviors. One 1-h interview	Linkage with SUD treatment within 90 days	3 months	SBCM (*n* = 222) was more effective in improving linkage compared to CG (*n* = 230), 55.0 vs. 38.7%, respectively (*p* < 0.01). SBCM improved linkage more than MI did (55.0 vs. 44.7%, *p* < 0.05). MI (*n* = 226) was not significantly more effective in improving linkage than in CG (44.7 vs. 38.7%; *p* > 0.05). The three trial groups differed only slightly on the client characteristics that predicted linkage with treatment
Saleh et al. (34), USA	Alcohol or drug abuse	*N* = 627	Usual care in treatment centers	12 months of CM services in community non-profit substance abuse treatment centers	Number of hospitalization days, number of ER visits, number of physician visits. Study 31: legal, employment, psychiatric improvements	3, 6, and 12 months	IGs showed decrease of the usage of mental health services. However, hospital usage, ER visits, and access to physicians were increased in IGs. The short duration of CM services was expected to increase the use of access outcomes. Study 32: legal, employment, and psychiatric improvements
Saleh et al. (35)		IG: *n* = 437 (treatment agency: *n* = 167, social service agency: *n* = 160, telecom CM: *n* = 147); CG: *n* = 188					
Scott et al. (33), USA	Substance abuse clients who used alcohol or other drugs in the past 6 months and who were enrolled in one of nine community substance abuse treatment facilities	*N* = 692 IG: *n* = 344; CG: *n* = 348	Usual care in community	CM services over a 22-month period: assessment, referral services, client advocacy, counseling, and follow-up treatment	Treatment retention, show rates to treatment		IG was significantly more likely to show response to treatment than CG. No differences found in dose (amount or length of substance abuse treatment services) in both IG and CG
Siegal et al. (25), USA	Veterans seeking treatment for substance abuse problems	*N* = 632 CM: *n* = 313; CG: *n* = 319	CG: no CM group	Veterans in the inpatient component participate in three phases lasting a total of 28 days. Outpatients attend 10 weeks of sessions involving education about substance abuse problems and group therapy sessions designed to assist in achieving abstinence. Both inpatient and outpatient clients are referred to an aftercare service upon completion of primary treatment. The clients in the IG received help for strengths assessment, identifying goals, and, if appropriate, accompaniment on job search	Substance use (ASI), psychosocial functioning, employment outcomes	6 months	All clients showed significant improvement in employment outcomes, an increase of 6 days worked (*p* < 0.01) in the last 30 days before the 3-month follow-up. SBCM reported 3.5 additional days worked compared to non-case-managed clients. There was a positive relationship between improved employment functioning and improvement in other life areas
Slesnick and Erdem (32), USA	Substance-abusing homeless mothers with a 2- to 6-year-old child	*N* = 60 IG: *n* = 30; CG: *n* = 30	Usual care in community	Ecologically based treatment with CM services. The mothers were housed in apartments of their choosing and received 3 months of utility and rental assistance. CM services for 6 months, focusing on basic needs (i.e., referrals to food pantries); assisting, obtaining government entitlements; employment; connecting to social services; providing referrals and/or transportation to appointments. Average of 23.1 sessions in 6 months	Substance use, retention rate, independent living days	3, 6, and 9 months	Mothers receiving ecologically based treatment showed a high retention rate on treatment, a faster decline in alcohol use (*p* < 0.05), and a faster increase in their independent living days (*p* < 0.001). Furthermore, with supportive services, two-thirds of women were successful in maintaining their apartments 6 months after rental assistance ended. However, no treatment effects were found in drug use (*p* > 0.05)
Strathdee et al. (26), USA	Clients of the Baltimore Needle Exchange Program who sought drug abuse treatment	*N* = 245 IG: *n* = 128; CG: *n* = 117	Passive referral [voucher printed with date, time, and location for intake appointment (of opioid agonist) at the drug-treatment program]	SBCM: engagement, strengths assessment, personal case planning, resource acquisition. The duration and frequency of CM contacts were client-driven, based on individual desires and needs	Intake appointment for opioid agonist therapy within 7 days	7 days	In a multivariate “intention-to-treat” model (i.e., ignoring the amount of CM actually received), those randomized to CM were more likely to enter treatment within 7 days (40 vs. control: 26%, *p* = 0.03). Additional “as-treated” analyses revealed that participants who received 30 min or more of CM within 7 days were 33% more likely to enter treatment. The active ingredient of CM activities was provision of transportation

*CM, case management; SUD, substance use disorder; IG, intervention group; CG, control group; PCM, probation case management; TAU, treatment as usual; ASI, Addiction Severity Index; BDI, Beck Depression Inventory; BSI, Brief Symptom Inventory; AUDIT, Alcohol Use Disorders Identification Test; AUDRUG, Drug Use Identification Test; SIP, Short Index of Problems; ICM, intensive case management; CCM, coordinated care management; SR, standard referral; TCM, transitional case management; SBCM, strengths-based case management; MI, motivational interviewing; ER, emergency room*.

The names of CM interventions varied in different studies. They were labeled “intensive,” “community,” or “assertive CM” (20–22); “strengths-based” (23–26), “clinical” (27), and “transitional CM” (28); or “coordinated care management” (29) and “probation CM” (30). Although the names and interventions varied, certain common characteristics could be found. CM services were conducted by case managers with a professional background in nursing, social work, or mental health care (22). CM services were delivered mainly in the patients’ communities and not at the treatment center or hospital (20–22). The length of interventions varied from 1 month (25) to 3 years (20), although 6 months to 1 year was the most common. The intensity of the CM intervention was rarely noted.

### Study Populations

In some studies, the population had SUD and no further differentiation was made, whereas other studies considered specific subgroups such as patients in methadone programs (27), women with SUD (22, 31, 32), and participants with court judgments who were either incarcerated or in court-ordered treatments (23, 28, 30). Most studies were done in the United States, except for the one by Prendergast et al. (28) in Canada and Lindhal et al. (23) in Sweden.

### Outcome Measures

The most frequently used outcome measures were change in drug or alcohol use, as well as adherence to SUD treatment (frequently measured in attendance rates) and linkage to other health-care providers. The other important outcome measures were health-care use in terms of days of hospitalization, emergency ward visits, or health costs. On a more general level, some studies measured global functioning; employment rates; reduction of social, legal, and family problems; and client satisfaction. Two studies concerning incarcerated or court-ordered individuals also used the number of post-enrollment arrests as an outcome measure (28, 30).

Most studies considered only SUD as an inclusion criterion. Surprisingly, the importance of comorbid mental health disorders or high service use was not defined as an inclusion criterion in most studies. Slesnick and Erdam’s study (32) included only homeless mothers, and Morgenstern et al.’s studies (22, 29, 31) analyzed patients receiving welfare [in one study (22), only women were included]. Only Essock et al.’s study (20) used high service use, severe comorbid mental health disorder, unstable housing, and poor living skills as inclusion criteria. Some studies even excluded psychotic disorders (22, 24), and Morgenstern et al.’s study (29) excluded patients who had been hospitalized more than once for mental health reasons in the past year.

### Effect of the Intervention

Only two studies (28, 30) did not find any additional value in CM when treating addicted patients. The other 12 papers found significant improvement of some or all the outcome measures. These improvements were not the same for each survey.

Five studies showed that substance use decreased (20, 22, 23, 27, 32), two papers (22, 26) showed that the likelihood of initiating SUD treatment increased, and four publications (22, 23, 27, 33) showed greater treatment retention when a case manager was involved in treatment. Four studies (23, 24, 33, 34) showed improved access to health care and/or linkage between health-care providers. One research showed fewer days spent in hospital (20) but others reported an increased number of days in hospital, which is explained by the higher treatment retention (34). Seven publications showed better global functioning, which was described as more employment days (25, 31, 35). This was further differentiated in Morgenstern et al.’s study of 2008 (29), which showed that women were more likely than men to find employment when assisted by CM, to have fewer legal (21, 35) and family problems (21), and to have better housing stability (32). Lindahl et al. measured very high patient satisfaction with the treatment and 100% treatment retention compared to TAU (23).

## Discussion

In most studies, significant improvements were reported in the outcome measures. Substance use decreased in only five papers (20, 22, 23, 27, 32), but treatment adherence and linkage between health-care providers seemed to improve in most surveys, which is an important issue for this population and one of the main aims of CM. Overall functioning improved in more than half of the studies, which is in general linked to higher life satisfaction.

The two publications (28, 30) that did not find significant improvements in one of the outcome measures were both performed with incarcerated or paroled patients. In Guydish et al.’s paper (30), the important factor was the limited face-to-face time. Only 53.6% of participants had seen their CM once or more during the first 6 months. For those participants who had seen their CM two or more times in the first 6 months, there was an improvement in substance use and social problems. This finding shows how important treatment intensity of CM is for the outcome. The other study with a negative outcome, by Prendergast et al. (28), showed no improvement in treatment participation for parolees with SUD who were receiving CM. However, this finding cannot be generalized, as the case manager’s adherence to the protocol and the intervention was not standardized. Moreover, the case manager seemed to have limited contact with the parolee and the parolees did not enroll voluntarily in the project.

The studies are heterogeneous in their clinical approach, which limits our ability to generalize specific implications for practice. Different types of populations with different risk levels seem to account for the variation in readmission rates (36). For example, the study populations varied in illness severity (comorbidities and service use), which would most likely have an effect on the study outcome. Some comorbid populations were also excluded from surveys. The CM model is a specific intervention that seems more useful to specific subgroups who are unable to use existing health-care services. A large number of patients find adequate health care in the usual care programs, as shown in research on CM interventions that involve psychiatric patients with psychosis. Patients who benefit from CM have been shown to be those with greater social and psychosocial needs, more psychiatric symptoms, and higher service use (37), with others not needing this specific intensive care (38). If CM is applied to a large group, the effect on a smaller subgroup would likely be diluted and not as visible in the outcome measures. It will be important to further specify these subgroups in future in order to refer patients to the appropriate programs.

The intensity of application of CM differs in these studies, which also limits our ability to generalize the effects (39). These examples show the importance of face-to-face time with patients, which can be managed only with small caseloads. This seems to have been a limiting factor in the two studies (28, 30) mentioned above. In addition, the adherence to the model by the case manager and the voluntary participation of the patients seem to affect the outcome.

The majority of the studies were performed in the United States, which is a limiting factor in generalizing these findings to other countries such as those in Europe, where health systems differ in organization and funding. A further limitation of this research was that the data search was performed only on the PubMed database and possibly that unpublished negative studies were not taken into account. Furthermore, we did not differentiate between alcohol use disorder and other SUDs.

## Conclusion

Most of the analyzed studies showed improvement in the chosen outcome measures, although these varied in different studies. Treatment adherence mostly improved, but substance use decreased in only a third of the studies. Overall functioning improved in about half of the studies. The differences in chosen outcome measures make it difficult to compare the results. The type of intervention and intensity of treatment also varied.

The heterogeneity of these results might be linked to the different types of populations studied. The specific CM intervention seems to be helpful only for specific subpopulations with SUDs. Further studies are necessary to determine inclusion criteria for CM treatment for patients with SUD in order to orientate those most likely to benefit from this approach to the specific CM programs.

Only a few studies on this intervention and SUD have been published. Further research is needed to examine the effect of treatment intensity of the CM intervention. Longitudinal studies are also needed in Europe to ensure the effectiveness of these treatments.

## Author Contributions

LP, AM, and YK designed the strategy for the present review and drafted the manuscript. AM and LP searched for the references and read the manuscripts. LP, AM, and YK discussed the results. All authors reviewed the manuscript and helped with the final writing.

## Conflict of Interest Statement

The authors declare that the research was conducted in the absence of any commercial or financial relationships that could be construed as a potential conflict of interest.
